# Responses of *Pseudomonas aeruginosa* to antimicrobials

**DOI:** 10.3389/fmicb.2013.00422

**Published:** 2014-01-08

**Authors:** Yuji Morita, Junko Tomida, Yoshiaki Kawamura

**Affiliations:** Department of Microbiology, School of Pharmacy, Aichi Gakuin University, NagoyaJapan

**Keywords:** *pseudomonas aeruginosa*, anti-bacterial agents, stress responses, multidrug efflux systems, biofilms

## Abstract

Infections caused by *Pseudomonas aeruginosa* often are hard to treat; inappropriate chemotherapy readily selects multidrug-resistant *P. aeruginosa*. This organism can be exposed to a wide range of concentrations of antimicrobials during treatment; learning more about the responses of *P. aeruginosa* to antimicrobials is therefore important. We review here responses of the bacterium *P. aeruginosa* upon exposure to antimicrobials at levels below the inhibitory concentration. Carbapenems (e.g., imipenem) have been shown to induce the formation of thicker and more robust biofilms, while fluoroquinolones (e.g., ciprofloxacin) and aminoglycosides (e.g., tobramycin) have been shown to induce biofilm formation. Ciprofloxacin also has been demonstrated to enhance the frequency of mutation to carbapenem resistance. Conversely, although macrolides (e.g., azithromycin) typically are not effective against *P. aeruginosa* because of the pseudomonal outer-membrane impermeability and efflux, macrolides do lead to a reduction in virulence factor production. Similarly, tetracycline is not very effective against this organism, but is known to induce the type-III secretion system and consequently enhance cytotoxicity of *P. aeruginosa* *in vivo*. Of special note are the effects of antibacterials and disinfectants on pseudomonal efflux systems. Sub-inhibitory concentrations of protein synthesis inhibitors (aminoglycosides, tetracycline, chloramphenicol, etc.) induce the MexXY multidrug efflux system. This response is known to be mediated by interference with the translation of the leader peptide PA5471.1, with consequent effects on expression of the PA5471 gene product. Additionally, induction of the MexCD-OprJ multidrug efflux system is observed upon exposure to sub-inhibitory concentrations of disinfectants such as chlorhexidine and benzalkonium. This response is known to be dependent upon the AlgU stress response factor. Altogether, these biological responses of *P. aeruginosa* provide useful clues for the improvement and optimization of chemotherapy in order to appropriately treat pseudomonal infections while minimizing the emergence of resistance.

## INTRODUCTION

*Pseudomonas aeruginosa,* a motile, non-fermenting Gram-negative bacterium, is an opportunistic pathogen implicated in respiratory infections, urinary tract infections, gastrointestinal infections, keratitis, otitis media, and bacteremia in patients with compromised host defenses [e.g., cancer, burn, HIV, and cystic fibrosis (CF)]. These infections often result in significant morbidity and mortality. In the 21st century, when the life expectancy of highly susceptible immunocompromised groups has been extended in most countries, *P. aeruginosa* plays an increasingly prominent role in hospital infections.

This organism is a ubiquitous and metabolically versatile microbe that flourishes in many environments. The bacterium grows under both aerobic and anaerobic conditions, and possesses numerous virulence factors that contribute to its pathogenesis (Schurek et al., 2012). Moreover *P. aeruginosa* possesses an intrinsic resistance to many antimicrobials because of the bacterium’s outer-membrane barrier, the presence of multidrug efflux transporters, and endogenous antimicrobial inactivation (Poole, 2011). Although anti-pseudomonas agents (e.g., carbapenems) have been discovered and developed, *P. aeruginosa* readily acquires resistance to individual agents via chromosomal mutations and lateral gene transfer (Poole, 2011).

*Pseudomonas aeruginosa* possesses multifactorial mechanisms of responses and resistance to antimicrobials. While antimicrobials were originally developed and used to kill bacteria, recent work reveals that the biological functions of antibiotics are not limited to bactericidal (killing) or bacteriostatic (growth inhibition) effects (Linares et al., 2006; Aminov, 2013). The most likely function of antibiotics in natural ecosystems is in intercellular “signaling,” with specific consequences on the collective behavior of the bacterial population (Linares et al., 2006; Aminov, 2013). Improved genetic tools and cutting-edge technologies (e.g., DNA microarrays) have revolutionized our understanding of microbial physiology (Wecke and Mascher, 2011). Here, we summarize and discuss how *P. aeruginosa* responds to various antimicrobials and survives against its competitors.

## RESPONSES TO β-LACTAMS

β-lactams bind to cell wall transpeptidases [penicillin binding proteins (PBPs)], blocking an important step in peptidoglycan biosynthesis (Poole, 2004). Penicillins (e.g., ticarcillin, piperacillin), cephalosporins (e.g., ceftazidime, cefepime), monobactams (e.g., aztreonam), and carbapenems (e.g., imipenem, meropenem, and doripenem) are commonly used to treat pseudomonal infections. *P. aeruginosa* is intrinsically resistant to most β-lactams due to the interplay of the inducible β-lactamase AmpC and the resistance nodulation cell division (RND) multidrug efflux systems (e.g., MexAB-OprM; Masuda et al., 1999). Benzyl-penicillins (e.g., amoxicillin) and narrow-spectrum cephalosporins are labile to hydrolysis and are strong inducers of *ampC*, leading to antibiotic degradation, whereas ureidopenicillins (e.g., piperacillin) and extended-spectrum cephalosporins are labile but are weak inducers of *ampC* (Livermore, 1995). The *mexAB-oprM* operon is constitutively expressed in wild-type cells under usual laboratory conditions, where the operon contributes to *P. aeruginosa*’s intrinsic resistance to most β-lactams (except for imipenem) and many other antimicrobial agents, including quinolones, tetracycline, chloramphenicol, and macrolides (Morita et al., 2001). Blocking of *dacB*-encoded non-essential PBP4 determines a highly efficient and complex β-lactam resistance response, triggering the overproduction of AmpC and the specific activation of the CreAB (BlrAB) two-component regulator (Moya et al., 2009).

Carbapenems are an important class of anti-pseudomonal β-lactams. Carbapenems are strong inducers that are marginally labile (imipenem) or effectively stable (meropenem; Livermore, 1995). Imipenem also has been shown to strongly induce *ampC* gene expression in biofilms (Bagge et al., 2004). In addition, *P. aeruginosa* biofilms exposed to imipenem exhibit elevated expression of genes coding for alginate biosynthesis, causing thicker and more robust biofilms (Bagge et al., 2004).

Ceftazidime, a PBP3 inhibitor, does not induce *ampC* gene expression, but is rather a substrate of AmpC. Ceftazidime impacts the transcription of a large number of genes in *P. aeruginosa,* including those encoding the SOS response repressor LexA-like proteins, causing induced mutagenesis and decreasing ciprofloxacin toxicity (Blazquez et al., 2006). In addition, this antimicrobial shows quorum sensing (QS) inhibitory activity, decreasing the production of a range of QS-regulated virulence factors, in contrast to piperacillin, another PBP3 inhibitor (Skindersoe et al., 2008) These results imply that the QS inhibitory activity of ceftazidime likely is not PBP3-dependent (Skindersoe et al., 2008).

## RESPONSES TO FLUOROQUINOLONES

Fluoroquinolones, particularly ciprofloxacin, are commonly used for the treatment of *P. aeruginosa* infections (Poole, 2011). This class of agents interacts with complexes composed of DNA and either of the two target enzymes, DNA gyrase and/or topoisomerase IV. Primary intrinsic resistance of the wild-type *P. aeruginosa* to fluoroquinolones is due to MexAB-OprM as well as to MexXY-OprM (Morita et al., 2001). The four RND-type multidrug efflux pumps (MexAB-OprM, MexCD-OprJ, MexEF-OprN, and MexXY-OprM) are well recognized as significant determinants of fluoroquinolone resistance in lab and clinical isolates (Poole, 2011), although there are several additional chromosomally encoded efflux pumps that are able to recognize fluoroquinolones (e.g., MexHI-OpmD, MexVW-OprM, the NorM ortholog; Morita et al., 1998; Li et al., 2003; Sekiya et al., 2003; He et al., 2004).

The formation of static biofilms increases when *P. aeruginosa* cells are incubated in the presence of sub-inhibitory concentrations of ciprofloxacin, tobramycin, or tetracycline, while no such sub-inhibitory effect is detected with members of other antibiotic classes, such as carbenicillin, chloramphenicol, or polymyxin (Hoffman et al., 2005; Linares et al., 2006). However, in the presence of a sub-inhibitory concentration of ciprofloxacin (but not of tetracycline or tobramycin), *P. aeruginosa* shows a reduction in swimming and swarming, both of which are important systems of bacterial motility and probably related to the pathogenic process in CF patients (Linares et al., 2006).

Transcriptional responses of *P. aeruginosa* to sub-inhibitory and inhibitory concentrations of ciprofloxacin demonstrate the induction or repression of 100s of genes (Brazas and Hancock, 2005; Cirz et al., 2006; Brazas et al., 2007). Surprisingly, genes for bacteriophage-like pyocins are up-regulated and mediate fluoroquinolone susceptibility (Brazas and Hancock, 2005). At least one-third of up-regulated genes occur in regulons that are likely controlled by LexA-like SOS response repressor proteins in response to inhibitory concentrations of ciprofloxacin, while down-regulated genes appear to involve virtually every facet of cellular metabolism (Cirz et al., 2006; Brazas et al., 2007). The Lon protease modulates SOS response and consequently ciprofloxacin susceptibility (Breidenstein et al., 2012).

The overall pattern of expression of the DNA replication enzymes suggests a shift from canonical DNA replication enzymes to inducible polymerases in response to inhibitory ciprofloxacin concentrations (Cirz et al., 2006). These inhibitory concentrations of ciprofloxacin create selection pressure in favor of mutants with increased *ampC* expression (Wolter et al., 2007), while sub-inhibitory levels of ciprofloxacin or ofloxacin enhance the frequency of mutation to carbapenem (especially meropenem) resistance (Tanimoto et al., 2008).

## RESPONSES TO AMINOGLYCOSIDES

Aminoglycosides bind to the 30S ribosomal subunit and interfere with protein synthesis, causing mistranslation and ultimately cell death without lysis (Davis, 1987). APH(3′)-IIb, a chromosomal *aphA*-encoded aminoglycoside phosphoryltransferase, is likely responsible for the general non-susceptibility of *P. aeruginosa* to kanamycin (Hachler et al., 1996), and APH(3′)-II predominates in clinical isolates resistant to kanamycin (Poole, 2005). Anti-pseudomonas aminoglycosides (e.g., amikacin, gentamicin, and tobramycin) therefore can be used in the treatment of *P. aeruginosa* infections (Poole, 2005). Aminoglycoside uptake and subsequent action within bacterial cells is a complex process that involves Lipopolysaccharides (LPS) binding and outer-membrane permeation, cytoplasmic membrane traversal driven by membrane potential, and ribosome disruption, leading to the production of membrane-damaging mistranslated polypeptides (Davis, 1987; Krahn et al., 2012). Primary intrinsic and adaptive resistance to aminoglycosides is due to the MexXY multidrug efflux system in laboratory and clinical isolates (Morita et al., 2012b). The antagonism of aminoglycosides by divalent cations Mg^2^^+^ and Ca^2^^+^ is well documented in *P. aeruginosa*, and occurs via a process that requires the MexXY multidrug efflux system (Mao et al., 2001; Morita et al., 2012a). In wild-type *P. aeruginosa* cells, the MexXY efflux system is inducible by sub-inhibitory concentrations of aminoglycoside- and ribosome-targeting antimicrobials (e.g., chloramphenicol and tetracycline), a process shown to be involved in expression of the PA5471 gene product [recently renamed ArmZ, for anti-repressor MexZ (Morita et al., 2006; Hay et al., 2013)] The PA5471 system also is inducible through interference via translation of the gene’s leader peptide, PA5471.1 (Morita et al., 2009). Very recently a clinical strain overproducing MexXY was reported also to harbor a 7-bp deletion in the coding sequence of the leader peptide involved in ribosome-dependent, translational attenuation of *PA5471* expression (Guénard et al., 2013).

Transcriptomic analyses confirm that aminoglycosides impact the expression of a myriad of genes (Kindrachuk et al., 2011). While prolonged exposure to sub-inhibitory concentrations of tobramycin causes increased levels of expression, predominantly of the *mexXY* efflux pump genes, the greatest increases in gene expression levels in response to lethal concentrations of tobramycin involve a number of *P. aeruginosa* heat shock genes (e.g., *htpG*, *ibpA*, *groES,* and *asrA*; Kindrachuk et al., 2011). Under these conditions, the likely intracellular ATP-dependent AsrA protease is noteworthy because of its modest positive impact on aminoglycoside resistance (Kindrachuk et al., 2011). The Lon protease also is inducible by aminoglycosides (Marr et al., 2007).

Sub-inhibitory concentrations of aminoglycosides, especially tobramycin, induce biofilm formation in *P. aeruginosa* (Hoffman et al., 2005). Notably, the aminoglycoside tobramycin also induces both swimming and swarming of *P. aeruginosa* (Linares et al., 2006). Also induced is the *aminoglycoside response regulator* (*arr*) gene, which is predicted to encode an inner membrane phosphodiesterase. The Arr substrate is cyclic di-guanosine monophosphate (c-di-GMP), a bacterial second messenger that regulates cell surface adhesiveness; c-di-GMP is essential for this induction and contributes to biofilm-specific aminoglycoside resistance (Hoffman et al., 2005).

## RESPONSES TO POLYMYXINS

Owing to the increased prevalence of multidrug-resistant *P. aeruginosa,* polymyxin B and colistin (also called polymyxin E), belonging to a family of antimicrobial cyclic oligopeptides, have returned to favor as a last-resort treatment option, although these agents have strong side effects (e.g., nephrotoxicity) with high incidence (Poole, 2011). Very recently, the polymyxin mutant prevention concentrations (MPCs) for *P. aeruginosa* were shown to be very high (≥64 μg/ml), even for susceptible isolates (i.e., with minimum inhibitory concentration (MIC) ranges of 1–2 μg/ml; Choi and Ko, 2013). In the MPC studies, mutation to polymyxin resistance apparently can result from a single mutation (Choi and Ko, 2013).

The mechanism of action of polymyxins involves an initial stage of interaction with the lipid A of the LPS, leading to self-promoted uptake of polymyxins across the membrane, followed by cell death (Zhang et al., 2000; Fernandez et al., 2013). The most common mechanism of resistance to polymyxin has been shown to arise from modification of LPS lipid A with 4-amino-L-arabinose, a process that has been seen both in *in vitro-*selected mutants and in CF isolates; other unknown mechanisms remain under investigation (Miller et al., 2011; Poole, 2011; Moskowitz et al., 2012). This modification is carried out by the products of the *arnBCADTEF*-*ugd* operon, otherwise known as *pmrHFIJKLM*-*ugd* (McPhee et al., 2003; Yan et al., 2007). The two-component ParRS regulator leads to the induction of the LPS modification operon in response to sub-inhibitory concentrations of polymyixin B and colistin (Fernandez et al., 2010).

Approximately 0.5% of genes showed significantly altered expression upon exposure to sub-inhibitory concentration of colistin (Cummins et al., 2009), a frequency that is no less dramatic than that seen with the other anti-pseudomonas agents (e.g., ceftazidime, ciprofloxacin, and tobramycin) described above. The most striking alterations were up-regulation of the *Pseudomonas* quinolone signal (PQS) biosynthetic genes such as the *pqsABCDE* operon, the phenazine biosynthetic operon, and the *arn* operon (Cummins et al., 2009).

## RESPONSES TO THE MAJOR HUMAN CATIONIC HOST DEFENSE PEPTIDE, LL-37

The major human cationic host defense peptide LL37 [a.k.a. hCAP-18, FALL-39, or cathelicidin antimicrobial peptide (CAMP)], a 37-amino-acid, 18-kDa peptide, is encoded by the cathelicidin gene (CAMP) and was originally identified in humans (Kosciuczuk et al., 2012). Sub-inhibitory concentration of LL-37 (1/4–1/128 of the MIC of 64 μg/ml) were shown to prevent biofilm formation by decreasing the attachment of *P. aeruginosa* cells, stimulating twitching motility, and influencing two major QS systems (Las and Rhl), leading to the down-regulation of genes essential for biofilm development (Overhage et al., 2008). Similar results were obtained using the bovine neutrophil peptide indolicidin, but no inhibitory effects on biofilm formation were detected using sub-inhibitory concentrations of the mouse peptide CRAMP (67% identical with LL-37), polymyxin B, or the bovine bactenecin homolog Bac2A (Overhage et al., 2008).

## RESPONSES TO MACROLIDES

Macrolides such as erythromycin and azithromycin are widely used antibiotics that block translation by binding to the 50S ribosomal subunit. *P. aeruginosa* cells are intrinsically resistant to macrolides when tested in standard broth culture; for instance, the MIC of erythromycin for *P. aeruginosa* PAO1 is about 512 μg/mL in Mueller-Hinton broth (e.g., Morita et al., 2001; Buyck et al., 2012). Nonetheless, low-dose macrolides such as azithromycin are effective treatments in patients with chronic lung infections (Jaffe et al., 1998; Kudoh et al., 1998). Even at concentrations (e.g., 2 μg/ml of azithromycin) far below the MIC, macrolides inhibit the QS circuitry of *P. aeruginsoa* strain PAO1, leading to a reduction in virulence factor production (Tateda et al., 2001). Low-dose azithromycin shows bactericidal activity for *P. aeruginosa* biofilms, but selects for *nfxB* mutants, which overproduce the MexCD-OprJ efflux pump (Mulet et al., 2009). Notably, the AmpC β-lactamase produced by *nfxB* mutants is protective in biofilm growth, although over expression of the MexCD-OprJ pump is known to impair *P. aeruginosa’*s intrinsic resistance, which is dependent on the MexXY/MexAB-OprM efflux pump and the AmpC (Mulet et al., 2011).

Genome-wide approaches have revealed the QS antagonistic activities of azithromycin (e.g., inhibition of QS, reduction of virulence factor production, and strong induction of type-III secretion systems; Nalca et al., 2006; Skindersoe et al., 2008). This modulation causes decreased expression of the genes encoding the MexAB-OprM efflux pump in *P. aeruginosa* (Sugimura et al., 2008). Azithromycin inhibits expression of the small RNAs *rsmY* and *rsmZ*, a process that depends on the GacA/Rsm signal transduction pathway; this pathway is known to positively control *P. aeruginosa* QS (Perez-Martinez and Haas, 2011). Both effects of azithromycin on QS (quorum factor-dependent virulence factor production and cell death) require azithromycin to interact with ribosomes (Kohler et al., 2007). The stationary-phase killing of azithromycin is further enhanced by the production of rhamnolipids, which likely facilitate macrolide uptake (Kohler et al., 2007). The mode of action of azithromycin *in vivo* also is demonstrated through mutations in 23S rRNA that confer azithromycin resistance in bacterial isolates of *P. aeruginosa* in chronically infected CF patients (Marvig et al., 2012). The clinical efficacy of macrolides in treating pseudomonal infections can be partially explained by the increased susceptibility of *P. aeruginosa* to these compounds in eukaryotic cell culture media and biological fluids, due to decreased *oprM* expression and increased outer-membrane permeability (Buyck et al., 2012).

## RESPONSES TO TETRACYCLINES AND CHLORAMPHENICOL

Tetracyclines are bacteriostatic antibiotics based on a hydroanthracene nucleus, which contains four fused rings. The class also includes glycylcyclines (e.g., tigecycline), a group of semisynthetic tetracycline derivatives containing a glycylamido substitution at position 9 (Yao and Moellering, 2011). Tetracyclines enter bacteria by an energy-dependent process and bind reversibly to the 30S ribosomal subunit, preventing the attachment of aminoacyl-tRNA to the ribosomal acceptor A-site in the RNA-ribosome complex (Yao and Moellering, 2011). *P. aeruginosa* is intrinsically resistant to tetracyclines and glycylcyclines due to the MexAB/MexXY efflux systems (Morita et al., 2001; Dean et al., 2003). Sub-inhibitory concentrations of tetracycline and tigecycline induce the MexXY RND efflux system via a mechanism dependent on the ribosomal inhibitor-inducible PA5471 gene product (Dean et al., 2003; Morita et al., 2006).

Tetracycline increases biofilm formation as well as ciprofloxacin and tobramycin, as described above (Linares et al., 2006). Surprisingly, incubation with tetracycline at concentrations (~1 μg/ml) that do not decrease the growth rate of *P. aeruginosa* increases the expression of type-III secretion system (T3SS) genes *exsA* and *exoS*, and the presence of tetracycline increases the cytotoxicity of *P. aeruginosa* by nearly 4-fold (Linares et al., 2006). The T3SS is a mechanism by which bacterial pathogens can deliver effectors directly into the cytoplasm of the eukaryotic host cell (Hauser, 2009). Expression of genes forming the T3SS regulon is triggered by ExaA (Hauser, 2009), a transcriptional activator that auto-regulates its own expression by a feedback mechanism (Hauser, 2009). ExoS corresponds to a T3SS-secreted toxin (Hauser, 2009).

Chloramphenicol is a bacteriostatic agent that inhibits protein synthesis by binding reversibly to the peptidyltransferase component of the 50S ribosomal subunit and preventing the transpeptidation process of peptide chain elongation (Yao and Moellering, 2011). *P. aeruginosa* is usually intrinsically resistant to chloramphenicol, in part due to the MexAB-OprM efflux system (Morita et al., 2001). In addition, sub-inhibitory concentrations of chloramphenicol induce the MexXY efflux system via a mechanism dependent on the ribosomal inhibitor-inducible PA5471 gene product (Morita et al., 2006). This effect is reminiscent of the induction of the MexEF-OprN efflux system (via the MexT activator) in response to chloramphenicol and nitrosative stress (Fetar et al., 2011). Chloramphenicol is a nitro-aromatic antimicrobial and resembles a nitrosative stress product (Fetar et al., 2011).

## RESPONSE TO BIOCIDES

*Pseudomonas aeruginosa* also has been reported to contaminate disinfectants (e.g., chlorhexidine, benzalkonium, and triclosan) in hospital or other such environments, thereby compromising the disinfectant’s ability to reduce or eliminate bacterial contamination. Chlorhexidine and benzalkonium are a cationic biguanide and a nitrogen-based quaternary ammonium compound, respectively. These biocides function by affecting the cell membrane, resulting in lysis and the loss of cytoplasmic material (Poole, 2002). The RND-type MexCD-OprJ multidrug efflux pump is induced by sub-inhibitory concentrations of disinfectants such as benzalkonium chloride or chlorhexidine (Morita et al., 2003), a process that is dependent upon the AlgU stress response factor (Fraud et al., 2008). Global transcriptome response to chlorhexidine includes up-regulation of the *mexCD-oprJ* and *oprH-phoPQ* operons and down-regulation of genes encoding proteins involved in membrane transport, oxidative phosphorylation, electron transport, and DNA repair (Nde et al., 2009). A *P. aeruginosa* variant highly adapted to benzalkonium showed increased resistance to fluoroquinolones, owing to mutations in the quinolone resistance-determining region of *gyrA* and to mutations in genes (*mexR* and *nfxB*) that encode repressors of *mexAB-oprM* and *mexCD-oprJ,* respectively (Mc Cay et al., 2010). Development of chlorhexidine-tolerant sub-populations in *P. aeruginosa* biofilms also is dependent on the *mexCD-oprJ* genes (Chiang et al., 2012).

Triclosan specifically inhibits fatty acid synthesis through inhibition of bacterial enoyl–acyl carrier protein reductase, although *P. aeruginosa* is intrinsically resistant to triclosan due to the structure of the pseudomonal FabV protein (a triclosan-resistant enoyl–acyl carrier protein reductase) and active efflux. This innate resistance stems from at least five RND efflux pumps, including MexAB-OprM (Mima et al., 2007; Zhu et al., 2010). In *P. aeruginosa* mutant cells lacking the *mexAB-oprM* genes, sub-inhibitory concentrations of triclosan lead to alterations in the expression of almost half the genome, with 28% of genes being significantly up-regulated and 16% being significantly down-regulated (Chuanchuen and Schweizer, 2012). QS-regulating genes are among the most strongly down-regulated, and surprisingly, iron homeostasis is completely blocked in triclosan-exposed cells, thus mimicking conditions with excess iron (Chuanchuen and Schweizer, 2012).

## SUMMING UP AND FURTHER PROSPECTS

The primary biological responses of *P. aeruginosa* to sub-inhibitory concentrations of antimicrobials are summarized in **Table 1**. Interestingly, clinically useful anti-pseudomonas agents (carbapenems, fluoroquinolones, and aminoglycosides) are known to efficiently kill *P. aeruginosa* planktonic cells when used properly, but lead to more severe biofilm development upon exposure at sub-inhibitory concentrations. This pattern means that optimization of anti-pseudomonas chemotherapy is critical; in the absence of such optimization, chemotherapy fails to treat the infection and readily selects multidrug-resistant *P. aeruginosa*. By contrast, macrolides (which are not effective against *P. aeruginosa* planktonic cells because of the planktonic cells’ intrinsic resistance) provide activity that is antagonistic to QS, thereby reducing pseudomonal virulence. These findings regarding responses to antimicrobial agents suggest a route toward conquering *P. aeruginosa* infections. For example, macrolides have been shown to augment the *in vitro* activity of anti-pseudomonas agents against biofilms (Lutz et al., 2012).

**Table 1:** Biological responses of *P. aeruginosa* exposed to various antimicrobials at levels below the inhibitory concentrations.

Antimicrobials	Biological responses	References
β-lactams	Induction of the AmpC β-lactamase(some β-lactams are inducers, but others are not)	Livermore (1995)
Carbapenems	Formation of thicker and more robust biofilms(induction of alginate biosynthesis)	Bagge etal. (2004)
Ceftazidime	Induction of mutagenesis and decreasing ciprofloxacin toxicity	Blazquez etal. (2006)
	Inhibition of quorum sensing	Skindersoe etal. (2008)
Fluoroquinolones	Induction of biofilm formation	Linares etal. (2006)
	Reduction in swimming and swarming	Linares etal. (2006)
	Induction of SOS response	Brazas and Hancock (2005)
	Up-regulation of the bacteriophage-like pyocins	Brazas and Hancock (2005)
	Shift from canonical DNA replication enzymes to inducible polymerases	Cirz etal. (2006)
	Enhancement of mutation frequency to β-lactam resistance.	Wolter etal. (2007), Tanimoto etal. (2008)
Protein synthesis inhibitors	Induction of the MexXY efflux system	Morita etal. (2012b)
Aminoglycosides	Induction of heat shock genes	Kindrachuk etal. (2011)
	Induction of biofilm formation	Hoffman etal. (2005)
	Induction of swimming and swarming	Linares etal. (2006)
	Induction of the Lon protease	Marr etal. (2007)
Macrolides	Quorum sensing antagonistic activity(reduction in virulence factor)	Tateda etal. (2001)
	Induction of the T3SS	Nalca etal. (2006)
	Down-regulation of the MexAB-OprM pump	Sugimura etal. (2008)
Tetracycline	Induction of biofilm formation	Linares etal. (2006)
	Induction of the T3SS and cytotoxicity	Linares etal. (2006)
Chloramphenicol	Induction of the MexEF-OprN efflux pump	Fetar etal. (2011)
Polymyxins	Modification of LPS lipid A with 4-amino-L-arabinose	Fernandez etal. (2010)
	Up-regulation of the PQS biosynthetic genes	Cummins etal. (2009)
Chlorhexidine	Induction of the MexCD-OprJ efflux pump	Morita etal. (2003)
	Induction of the oprH-phoPQ operon	Nde etal. (2009)

We are aware of potential molecular targets for novel anti-pseudomonas agents, including essential genes (Morita et al., 2010). Novel class anti-pseudomonas agents should be expected to minimize a severe situation in which few agents are effective against these organisms. However, it is a much more difficult task to develop novel class antimicrobials for *P. aeruginosa* because of the presence of low membrane permeability and the RND multidrug efflux pumps. In addition this organism has the ability to adapt to various stresses, including sub-inhibitory antimicrobial exposure, by recruiting antimicrobial resistance mechanisms, notably that of RND efflux systems such as the MexXY system. While many laboratories are currently screening, so far no efflux pump inhibitors have been made available for clinical settings. Screening for novel antibacterial agents, including efflux pump inhibitors, is currently in progress in many laboratories, including our own.

## Conflict of Interest Statement

The authors declare that the research was conducted in the absence of any commercial or financial relationships that could be construed as a potential conflict of interest.

## References

[B1] Aminov R. I. (2013). Biotic acts of antibiotics. *Front. Microbiol.* 4: 241 10.3389/fmicb.2013.00241 PMC3746177 23966991

[B2] Bagge N. Schuster M. Hentzer M. Ciofu O. Givskov M. Greenberg E. P. (2004). *Pseudomonas aeruginosa* biofilms exposed to imipenem exhibit changes in global gene expression and beta-lactamase and alginate production. *Antimicrob. Agents Chemother.* 48 1175–1187 10.1128/AAC.48.4.1175-1187.2004 15047518 PMC375275

[B3] Blazquez J. Gomez-Gomez J. M. Oliver A. Juan C. Kapur V. Martin S. (2006). PBP3 inhibition elicits adaptive responses in *Pseudomonas aeruginosa*. *Mol. Microbiol.* 62 84–99 10.1111/j.1365-2958.2006.05366.x 16956383

[B4] Brazas M. D. Breidenstein E. B. Overhage J. Hancock R. E. (2007). Role of lon, an ATP-dependent protease homolog, in resistance of *Pseudomonas aeruginosa* to ciprofloxacin. *Antimicrob. Agents Chemother.* 51 4276–4283 10.1128/AAC.00830-07 17893152 PMC2167996

[B5] Brazas M. D. Hancock R. E. (2005). Ciprofloxacin induction of a susceptibility determinant in *Pseudomonas aeruginosa*. *Antimicrob. Agents Chemother.* 49 3222–3227 10.1128/AAC.49.8.3222-3227.2005 16048929 PMC1196232

[B6] Breidenstein E. B. Bains M. Hancock R. E. (2012). Involvement of the lon protease in the SOS response triggered by ciprofloxacin in *Pseudomonas aeruginosa* PAO1. *Antimicrob. Agents Chemother.* 56 2879–2887 10.1128/AAC.06014-11 22450976 PMC3370746

[B7] Buyck J. M. Plesiat P. Traore H. Vanderbist F. Tulkens P. M Van Bambeke F. (2012). Increased susceptibility of *Pseudomonas aeruginosa* to macrolides and ketolides in eukaryotic cell culture media and biological fluids due to decreased expression of oprM and increased outer-membrane permeability. *Clin. Infect. Dis.* 55 534–542 10.1093/cid/cis473 22573850

[B8] Chiang W. C. Pamp S. J. Nilsson M. Givskov M. Tolker-Nielsen T. (2012). The metabolically active subpopulation in *Pseudomonas aeruginosa* biofilms survives exposure to membrane-targeting antimicrobials via distinct molecular mechanisms. *FEMS Immunol. Med. Microbiol.* 65 245–256 10.1111/j.1574-695X.2012.00929.x 22251216

[B9] Choi M. J. Ko K. S. (2013). Mutant prevention concentrations of colistin for *Acinetobacter baumannii, Pseudomonas aeruginosa and Klebsiella pneumoniae* clinical isolates. *J. Antimicrob. Chemother*. 69 275–277 10.1093/jac/dkt315 23997018

[B10] Chuanchuen R. Schweizer H. P. (2012). Global transcriptional responses to triclosan exposure in *Pseudomonas aeruginosa*. *Int. J. Antimicrob. Agents* 40 114–122 10.1016/j.ijantimicag.2012.04.008 22704809

[B11] Cirz R. T. O’Neill B. M. Hammond J. A. Head S. R. Romesberg F. E. (2006). Defining the *Pseudomonas aeruginosa* SOS response and its role in the global response to the antibiotic ciprofloxacin. *J. Bacteriol.* 188 7101–7110 10.1128/JB.00807-06 17015649 PMC1636241

[B12] Cummins J. Reen F. J. Baysse C. Mooij M. J O’Gara F. (2009). Subinhibitory concentrations of the cationic antimicrobial peptide colistin induce the Pseudomonas quinolone signal in *Pseudomonas aeruginosa*. *Microbiology* 155 2826–2837 10.1099/mic.0.025643-0 19477905

[B13] Davis B. D. (1987). Mechanism of bactericidal action of aminoglycosides. *Microbiol. Rev.* 51 341–350 3312985 10.1128/mr.51.3.341-350.1987 PMC373115

[B14] Dean C. R. Visalli M. A. Projan S. J. Sum P. E. Bradford P. A. (2003). Efflux-mediated resistance to tigecycline (GAR-936) in *Pseudomonas aeruginosa* PAO1. *Antimicrob. Agents Chemother.* 47 972–978 10.1128/AAC.47.3.972-978.2003 12604529 PMC149306

[B15] Fernandez L. Alvarez-Ortega C. Wiegand I. Olivares J. Kocincova D. Lam J. S. (2013). Characterization of the polymyxin B resistome of *Pseudomonas aeruginosa*. *Antimicrob. Agents Chemother.* 57 110–119 10.1128/AAC.01583-12 23070157 PMC3535977

[B16] Fernandez L. Gooderham W. J. Bains M. McPhee J. B. Wiegand I. Hancock R. E. (2010). Adaptive resistance to the “last hope” antibiotics polymyxin B and colistin in *Pseudomonas aeruginosa* is mediated by the novel two-component regulatory system ParR-ParS. *Antimicrob. Agents Chemother.* 54 3372–3382 10.1128/aac.00242-210 20547815 PMC2916309

[B17] Fetar H. Gilmour C. Klinoski R. Daigle D. M. Dean C. R. Poole K. (2011). mexEF-oprN multidrug efflux operon of *Pseudomonas aeruginosa*: regulation by the MexT activator in response to nitrosative stress and chloramphenicol. *Antimicrob. Agents Chemother.* 55 508–514 10.1128/AAC.00830-10 21078928 PMC3028769

[B18] Fraud S. Campigotto A. J. Chen Z. Poole K. (2008). MexCD-OprJ multidrug efflux system of *Pseudomonas aeruginosa*: involvement in chlorhexidine resistance and induction by membrane-damaging agents dependent upon the AlgU stress response sigma factor. *Antimicrob. Agents Chemother.* 52 4478–4482 10.1128/AAC.01072-08 18838593 PMC2592872

[B19] Guénard S. Muller C. Monlezun L. Benas P. Broutin I. Jeannot K. (2013). Multiple mutations lead to MexXY/OprM-dependent aminoglycoside resistance in clinical strains of *Pseudomonas aeruginosa*. *Antimicrob. Agents Chemother.* 58 221–228 10.1128/AAC.01252-13 24145539 PMC3910787

[B20] Hachler H. Santanam P. Kayser F. H. (1996). Sequence and characterization of a novel chromosomal aminoglycoside phosphotransferase gene, aph (′)-IIb, in *Pseudomonas aeruginosa*. *Antimicrob. Agents Chemother.* 40 1254–1256 8723476 10.1128/aac.40.5.1254 PMC163301

[B21] Hauser A. R. (2009). The type III secretion system of *Pseudomonas aeruginosa*: infection by injection. *Nat. Rev. Microbiol.* 7 654–665 10.1038/nrmicro2199 19680249 PMC2766515

[B22] Hay T. Fraud S. Lau C. H. Gilmour C. Poole K. (2013). Antibiotic inducibility of the mexXY multidrug efflux operon of *Pseudomonas aeruginosa*: involvement of the MexZ anti-repressor ArmZ. *PLoS ONE* 8: e56858 10.1371/journal.pone.0056858 PMC3575510 23441219

[B23] He G. X. Kuroda T. Mima T. Morita Y. Mizushima T. Tsuchiya T. (2004). An H(+)-coupled multidrug efflux pump, PmpM, a member of the MATE family of transporters, from *Pseudomonas aeruginosa*. *J. Bacteriol.* 186 262–265 10.1128/JB.186.1.262-265.2004 14679249 PMC303449

[B24] Hoffman L. R. D’Argenio D. A. MacCoss M. J. Zhang Z. Jones R. A. Miller S. I. (2005). Aminoglycoside antibiotics induce bacterial biofilm formation. *Nature* 436 1171–1175 10.1038/nature03912 16121184

[B25] Jaffe A. Francis J. Rosenthal M. Bush A. (1998). Long-term azithromycin may improve lung function in children with cystic fibrosis. *Lancet* 351 420 10.1016/S0140-6736(05)78360-4 9482305

[B26] Kindrachuk K. N. Fernandez L. Bains M. Hancock R. E. (2011). Involvement of an ATP-dependent protease, PA0779/AsrA, in inducing heat shock in response to tobramycin in *Pseudomonas aeruginosa*. *Antimicrob. Agents Chemother.* 55 1874–1882 10.1128/AAC.00935-10 21357290 PMC3088180

[B27] Kohler T. Dumas J. L Van Delden C. (2007). Ribosome protection prevents azithromycin-mediated quorum-sensing modulation and stationary-phase killing of *Pseudomonas aeruginosa*. *Antimicrob. Agents Chemother.* 51 4243–4248 10.1128/AAC.00613-07 17876004 PMC2167979

[B28] Kosciuczuk E. M. Lisowski P. Jarczak J. Strzalkowska N. Jozwik A. Horbanczuk J. (2012). Cathelicidins: family of antimicrobial peptides. A review. *Mol. Biol. Rep.* 39 10957–10970 10.1007/s11033-012-1997-x 23065264 PMC3487008

[B29] Krahn T. Gilmour C. Tilak J. Fraud S. Kerr N. Lau C. H. (2012). Determinants of intrinsic aminoglycoside resistance in *Pseudomonas aeruginosa*. *Antimicrob. Agents Chemother.* 56 5591–5602 10.1128/AAC.01446-12 22908149 PMC3486610

[B30] Kudoh S. Azuma A. Yamamoto M. Izumi T. Ando M. (1998). Improvement of survival in patients with diffuse panbronchiolitis treated with low-dose erythromycin. *Am. J. Respir. Crit. Care Med.* 157 1829–1832 10.1164/ajrccm.157.6.9710075 9620913

[B31] Li Y. Mima T. Komori Y. Morita Y. Kuroda T. Mizushima T. (2003). A new member of the tripartite multidrug efflux pumps, MexVW-OprM, in *Pseudomonas aeruginosa*. *J. Antimicrob. Chemother.* 52 572–575 10.1093/jac/dkg390 12951344

[B32] Linares J. F. Gustafsson I. Baquero F. Martinez J. L. (2006). Antibiotics as intermicrobial signaling agents instead of weapons. *Proc. Natl. Acad. Sci. U.S.A.* 103 19484–19489 10.1073/pnas.0608949103 17148599 PMC1682013

[B33] Livermore D. M. (1995). beta-Lactamases in laboratory and clinical resistance. *Clin. Microbiol. Rev.* 8 557–584 8665470 10.1128/cmr.8.4.557 PMC172876

[B34] Lutz L. Pereira D. C. Paiva R. M. Zavascki A. P. Barth A. L. (2012). Macrolides decrease the minimal inhibitory concentration of anti-pseudomonal agents against *Pseudomonas aeruginosa* from cystic fibrosis patients in biofilm. *BMC Microbiol.* 12: 196 10.1186/1471-2180-12-196 PMC3485169 22958421

[B35] Mao W. Warren M. S. Lee A. Mistry A. Lomovskaya O. (2001). MexXY-OprM efflux pump is required for antagonism of aminoglycosides by divalent cations in *Pseudomonas aeruginosa*. *Antimicrob. Agents Chemother.* 45 2001–2007 10.1128/AAC.45.7.2001-2007.2001 11408215 PMC90592

[B36] Marr A. K. Overhage J. Bains M. Hancock R. E. (2007). The Lon protease of *Pseudomonas aeruginosa* is induced by aminoglycosides and is involved in biofilm formation and motility. *Microbiology* 153 474–482 10.1099/mic.0.2006/002519-0 17259618

[B37] Marvig R. L. Sondergaard M. S. Damkiaer S. Hoiby N. Johansen H. K. Molin S. (2012). Mutations in 23S rRNA confer resistance against azithromycin in *Pseudomonas aeruginosa*. *Antimicrob. Agents Chemother.* 56 4519–4521 10.1128/AAC.00630-12 22644032 PMC3421623

[B38] Masuda N. Gotoh N. Ishii C. Sakagawa E. Ohya S. Nishino T. (1999). Interplay between chromosomal beta-lactamase and the MexAB-OprM efflux system in intrinsic resistance to beta-lactams in *Pseudomonas aeruginosa*. *Antimicrob. Agents Chemother.* 43 400–402 9925544 10.1128/aac.43.2.400 PMC89089

[B39] Mc Cay P. H. Ocampo-Sosa A. A. Fleming G. T. (2010). Effect of subinhibitory concentrations of benzalkonium chloride on the competitiveness of *Pseudomonas aeruginosa* grown in continuous culture. *Microbiology* 156 30–38 10.1099/mic.0.029751-0 19815578

[B40] McPhee J. B. Lewenza S. Hancock R. E. (2003). Cationic antimicrobial peptides activate a two-component regulatory system, PmrA-PmrB, that regulates resistance to polymyxin B and cationic antimicrobial peptides in *Pseudomonas aeruginosa*. *Mol. Microbiol.* 50 205–217 10.1046/j.1365-2958.2003.03673.x 14507375

[B41] Miller A. K. Brannon M. K. Stevens L. Johansen H. K. Selgrade S. E. Miller S. I. (2011). PhoQ mutations promote lipid A modification and polymyxin resistance of *Pseudomonas aeruginosa* found in colistin-treated cystic fibrosis patients. *Antimicrob. Agents Chemother.* 55 5761–5769 10.1128/AAC.05391-11 21968359 PMC3232818

[B42] Mima T. Joshi S. Gomez-Escalada M. Schweizer H. P. (2007). Identification and characterization of TriABC-OpmH, a triclosan efflux pump of *Pseudomonas aeruginosa* requiring two membrane fusion proteins. *J. Bacteriol.* 189 7600–7609 10.1128/JB.00850-07 17720796 PMC2168734

[B43] Morita Y. Gilmour C. Metcalf D. Poole K. (2009). Translational control of the antibiotic inducibility of the PA5471 gene required for mexXY multidrug efflux gene expression in *Pseudomonas aeruginosa*. *J. Bacteriol.* 191 4966–4975 10.1128/JB.00073-09 19465646 PMC2715736

[B44] Morita Y. Kimura N. Mima T. Mizushima T. Tsuchiya T. (2001). Roles of MexXY- and MexAB-multidrug efflux pumps in intrinsic multidrug resistance of *Pseudomonas aeruginosa* PAO1. *J. Gen. Appl. Microbiol.* 47 27–32 10.2323/jgam.47.27 12483565

[B45] Morita Y. Kodama K. Shiota S. Mine T. Kataoka A. Mizushima T. (1998). NorM, a putative multidrug efflux protein, of *Vibrio parahaemolyticus* and its homolog in *Escherichia coli*. *Antimicrob. Agents Chemother.* 42 1778–1782 9661020 10.1128/aac.42.7.1778 PMC105682

[B46] Morita Y. Murata T. Mima T. Shiota S. Kuroda T. Mizushima T. (2003). Induction of mexCD-oprJ operon for a multidrug efflux pump by disinfectants in wild-type *Pseudomonas aeruginosa* PAO1. *J. Antimicrob. Chemother.* 51 991–994 10.1093/jac/dkg173 12654738

[B47] Morita Y. Narita S. Tomida J. Tokuda H. Kawamura Y. (2010). Application of an inducible system to engineer unmarked conditional mutants of essential genes of *Pseudomonas aeruginosa*. *J. Microbiol. Methods* 82 205–213 10.1016/j.mimet.2010.06.001 20538017

[B48] Morita Y. Sobel M. L. Poole K. (2006). Antibiotic inducibility of the MexXY multidrug efflux system of *Pseudomonas aeruginosa*: involvement of the antibiotic-inducible PA5471 gene product. *J. Bacteriol.* 188 1847–1855 10.1128/JB.188.5.1847-1855.2006 16484195 PMC1426571

[B49] Morita Y. Tomida J. Kawamura Y. (2012a). MexXY multidrug efflux system of *Pseudomonas aeruginosa*. *Front. Microbiol.* 3: 408 10.3389/fmicb.2012.00408 PMC3516279 23233851

[B50] Morita Y. Tomida J. Kawamura Y. (2012b). Primary mechanisms mediating aminoglycoside resistance in the multidrug-resistant *Pseudomonas aeruginosa* clinical isolate PA7. *Microbiology* 158 1071–1083 10.1099/mic.0.054320-0 22282519

[B51] Moskowitz S. M. Brannon M. K. Dasgupta N. Pier M. Sgambati N. Miller A. K. (2012). PmrB mutations promote polymyxin resistance of *Pseudomonas aeruginosa* isolated from colistin-treated cystic fibrosis patients. *Antimicrob. Agents Chemother.* 56 1019–1030 10.1128/AAC.05829-11 22106224 PMC3264203

[B52] Moya B. Dotsch A. Juan C. Blazquez J. Zamorano L. Haussler S. (2009). Beta-lactam resistance response triggered by inactivation of a nonessential penicillin-binding protein. *PLoS Pathog.* 5: e1000353 10.1371/journal.ppat.1000353 PMC2654508 19325877

[B53] Mulet X. Macia M. D. Mena A. Juan C. Perez J. L. Oliver A. (2009). Azithromycin in *Pseudomonas aeruginosa* biofilms: bactericidal activity and selection of nfxB mutants. *Antimicrob. Agents Chemother.* 53 1552–1560 10.1128/AAC.01264-08 19188376 PMC2663112

[B54] Mulet X. Moya B. Juan C. Macia M. D. Perez J. L. Blazquez J. (2011). Antagonistic interactions of *Pseudomonas aeruginosa* antibiotic resistance mechanisms in planktonic but not biofilm growth. *Antimicrob. Agents Chemother.* 55 4560–4568 10.1128/AAC.00519-11 21807976 PMC3186965

[B55] Nalca Y. Jansch L. Bredenbruch F. Geffers R. Buer J. Haussler S. (2006). Quorum-sensing antagonistic activities of azithromycin in *Pseudomonas aeruginosa* PAO1: a global approach. *Antimicrob. Agents Chemother.* 50 1680–1688 10.1128/AAC.50.5.1680-1688.2006 16641435 PMC1472232

[B56] Nde C. W. Jang H. J. Toghrol F. Bentley W. E. (2009). Global transcriptomic response of *Pseudomonas aeruginosa* to chlorhexidine diacetate. *Environ. Sci. Technol.* 43 8406–8415 10.1021/es9015475 19924977

[B57] Overhage J. Campisano A. Bains M. Torfs E. C. Rehm B. H. Hancock R. E. (2008). Human host defense peptide LL-37 prevents bacterial biofilm formation. *Infect. Immun.* 76 4176–4182 10.1128/IAI.00318-08 18591225 PMC2519444

[B58] Perez-Martinez I. Haas D. (2011). Azithromycin inhibits expression of the GacA-dependent small RNAs RsmY and RsmZ in *Pseudomonas aeruginosa*. *Antimicrob. Agents Chemother.* 55 3399–3405 10.1128/AAC.01801-10 21537014 PMC3122407

[B59] Poole K. (2002). Mechanisms of bacterial biocide and antibiotic resistance. *Symp. Ser. Soc. Appl. Microbiol.* 55S–64S 10.1046/j.1365-2672.92.5s1.8.x 12481829

[B60] Poole K. (2004). Resistance to beta-lactam antibiotics. *Cell. Mol. Life Sci.* 61 2200–2223 10.1007/s00018-004-4060-9 15338052 PMC11138534

[B61] Poole K. (2005). Aminoglycoside resistance in *Pseudomonas aeruginosa*. *Antimicrob. Agents Chemother.* 49 479–487 10.1128/AAC.49.2.479-487.2005 15673721 PMC547279

[B62] Poole K. (2011). *Pseudomonas aeruginosa*: resistance to the max. *Front. Microbiol.* 2: 65 10.3389/fmicb.2011.00065 PMC3128976 21747788

[B63] Schurek K. N. Breidenstein E. B. M Hancock R. E. W. (2012). “*Pseudomonas aeruginosa*: a persistent pathogen in cystic fibrosis and hospital-associated infections,” in *Antibiotic Discovery and Development,* Vol.1 eds Dougherty T. J. Pucci M. J. (New York: Springer) 679–715

[B64] Sekiya H. Mima T. Morita Y. Kuroda T. Mizushima T. Tsuchiya T. (2003). Functional cloning and characterization of a multidrug efflux pump, mexHI-opmD, from a *Pseudomonas aeruginosa* mutant. *Antimicrob. Agents Chemother.* 47 2990–2992 10.1128/AAC.47.9.2990-2992.2003 12937010 PMC182609

[B65] Skindersoe M. E. Alhede M. Phipps R. Yang L. Jensen P. O. Rasmussen T. B. (2008). Effects of antibiotics on quorum sensing in *Pseudomonas aeruginosa*. *Antimicrob. Agents Chemother.* 52 3648–3663 10.1128/AAC.01230-07 18644954 PMC2565867

[B66] Sugimura M. Maseda H. Hanaki H. Nakae T. (2008). Macrolide antibiotic-mediated down regulation of MexAB-OprM efflux pump expression in *Pseudomonas aeruginosa*. *Antimicrob. Agents Chemother.* 52 4141–4144 10.1128/AAC.00511-08 18676884 PMC2573159

[B67] Tanimoto K. Tomita H. Fujimoto S. Okuzumi K. Ike Y. (2008). Fluoroquinolone enhances the mutation frequency for meropenem-selected carbapenem resistance in *Pseudomonas aeruginosa*, but use of the high-potency drug doripenem inhibits mutant formation. *Antimicrob. Agents Chemother.* 52 3795–3800 10.1128/AAC.00464-08 18694945 PMC2565900

[B68] Tateda K. Comte R. Pechere J. C. Kohler T. Yamaguchi K Van Delden C. (2001). Azithromycin inhibits quorum sensing in *Pseudomonas aeruginosa*. *Antimicrob. Agents Chemother.* 45 1930–1933 10.1128/AAC.45.6.1930-1933.2001 11353657 PMC90577

[B69] Wecke T. Mascher T. (2011). Antibiotic research in the age of omics: from expression profiles to interspecies communication. *J. Antimicrob. Chemother.* 66 2689–2704 10.1093/jac/dkr373 21930574

[B70] Wolter D. J. Schmidtke A. J. Hanson N. D. Lister P. D. (2007). Increased expression of ampC in *Pseudomonas aeruginosa* mutants selected with ciprofloxacin. *Antimicrob. Agents Chemother.* 51 2997–3000 10.1128/AAC.00111-07 17517839 PMC1932541

[B71] Yan A. Guan Z. Raetz C. R. (2007). An undecaprenyl phosphate-aminoarabinose flippase required for polymyxin resistance in *Escherichia coli*. *J. Biol. Chem.* 282 36077–36089 10.1074/jbc.M706172200 17928292 PMC2613183

[B72] Yao J. D. C. Moellering J. R. (2011). “Antibacterial Agents,” in *Manual of Clinical Microbiology* 10th Edn Vol.1 ed. Versalovic J. (Washington: ASM Press)1043–1081

[B73] Zhang L. Dhillon P. Yan H. Farmer S. Hancock R. E. (2000). Interactions of bacterial cationic peptide antibiotics with outer and cytoplasmic membranes of *Pseudomonas aeruginosa*. *Antimicrob. Agents Chemother.* 44 3317–3321 10.1128/AAC.44.12.3317-3321.2000 11083634 PMC90199

[B74] Zhu L. Lin J. Ma J. Cronan J. E. Wang H. (2010). Triclosan resistance of *Pseudomonas aeruginosa* PAO1 is due to FabV, a triclosan-resistant enoyl-acyl carrier protein reductase. *Antimicrob. Agents Chemother.* 54 689–698 10.1128/AAC.01152-09 19933806 PMC2812149

